# Prevalence and antimicrobial resistance patterns of bacteria isolated from cerebrospinal fluid among children with bacterial meningitis in China from 2016 to 2018: a multicenter retrospective study

**DOI:** 10.1186/s13756-021-00895-x

**Published:** 2021-01-30

**Authors:** Xiaoshan Peng, Qingxiong Zhu, Jing Liu, Mei Zeng, Yue Qiu, Chunhui Zhu, Yibing Cheng, Yibo Zhou, Yi Xu, Minxia Chen, Zhengwang Wen, Yiping Chen, Rui Li, Jianning Tong, Qingwen Shan, Daojiong Lin, Shouye Wu, Zhiqiang Zhuo, Caihong Wang, Shiyong Zhao, Zhenghong Qi, Xiaofeng Sun, Bieerding Maihebuba, Chunmei Jia, Huiling Gao, Shuangjie Li, Yu Zhu, Chaomin Wan, the Collaborative Working Group of the Pediatric Subgroup of the China Society of Infectious Diseases

**Affiliations:** 1 Department of Pediatrics, West China Second Hospital, Sichuan University and Key Laboratory of Birth Defects and Related Diseases of Women and Children, Ministry of Education, No 20, 3rd Section of Renmin South Road, Chengdu, 610041 People’s Republic of China; 2 Department of Infectious Diseases, Children’s Hospital of Jiangxi Province, Nanchang, People’s Republic of China; 3 Department of Infectious Diseases, Hunan Children’s Hospital, Changsha, People’s Republic of China; 4 Department of Infectious Diseases, Children’s Hospital of Fudan University, Shanghai, People’s Republic of China; 5 Department of Emergency, Children’s Hospital Affiliated to Zhengzhou University (Henan Children’s Hospital), Zhengzhou, People’s Republic of China; 6 Department of General Pediatrics, Children’s Hospital Affiliated to Zhengzhou University (Henan Children’s Hospital), Zhengzhou, People’s Republic of China; 7 Department of Infectious Diseases, Guangzhou Women and Children’s Medical Center, Guangzhou, People’s Republic of China; 8 Department of Pediatrics, The Second Affiliated Hospital and Yuying Children’s Hospital of Wenzhou Medical University, Wenzhou, People’s Republic of China; 9 Department of Pediatrics, Gastroenterology and Infectious Diseases, Qingdao Women and Children’s Hospital, Qingdao, People’s Republic of China; 10 Department of Pediatrics, The First Affiliated Hospital of Guangxi Medical University, Nanning, People’s Republic of China; 11 Department of Infectious Diseases, Hainan Women and Children’s Medical Center, Haikou, People’s Republic of China; 12 Department of Infectious Diseases, Xiamen Children’s Hospital, Xiamen, People’s Republic of China; 13 Department of Infectious Diseases, Hangzhou Children’s Hospital, Hangzhou, People’s Republic of China; 14 Department of Infectious Diseases, The First Affiliated Hospital of Xinjiang Medical University, Ürümqi, People’s Republic of China; 15 Department of Pediatrics, The Fourth Hospital of Baotou, Baotou, People’s Republic of China; 16 Department of Pharmacy, The Fourth Hospital of Baotou, Baotou, People’s Republic of China; 17 Department of Hepatology, Hunan Children’s Hospital, No 86 Ziyuan Road, Changsha, 410000 People’s Republic of China

**Keywords:** Bacterial meningitis, Pediatric, Bacterial pathogens, Antimicrobial resistance

## Abstract

**Background:**

Pediatric bacterial meningitis (PBM) remains a devastating disease that causes substantial neurological morbidity and mortality worldwide. However, there are few large-scale studies on the pathogens causing PBM and their antimicrobial resistance (AMR) patterns in China. The present multicenter survey summarized the features of the etiological agents of PBM and characterized their AMR patterns.

**Methods:**

Patients diagnosed with PBM were enrolled retrospectively at 13 children’s hospitals in China from 2016 to 2018 and were screened based on a review of cerebrospinal fluid (CSF) microbiology results. Demographic characteristics, the causative organisms and their AMR patterns were systematically analyzed.

**Results:**

Overall, 1193 CSF bacterial isolates from 1142 patients with PBM were obtained. The three leading pathogens causing PBM were *Staphylococcus epidermidis* (16.5%), *Escherichia coli* (12.4%) and *Streptococcus pneumoniae* (10.6%). In infants under 3 months of age, the top 3 pathogens were *E. coli* (116/523; 22.2%), *Enterococcus faecium* (75/523; 14.3%), and *S. epidermidis* (57/523; 10.9%). However, in children more than 3 months of age, the top 3 pathogens were *S. epidermidis* (140/670; 20.9%), *S. pneumoniae* (117/670; 17.5%), and S*taphylococcus hominis* (57/670; 8.5%). More than 93.0% of *E. coli* isolates were sensitive to cefoxitin, piperacillin/tazobactam, cefoperazone/sulbactam, amikacin and carbapenems, and the resistance rates to ceftriaxone, cefotaxime and ceftazidime were 49.4%, 49.2% and 26.4%, respectively. From 2016 to 2018, the proportion of methicillin-resistant coagulase-negative Staphylococcus isolates (MRCoNS) declined from 80.5 to 72.3%, and the frequency of penicillin-resistant *S. pneumoniae* isolates increased from 75.0 to 87.5%. The proportion of extended-spectrum β-lactamase (ESBL)-producing *E. coli* fluctuated between 44.4 and 49.2%, and the detection rate of ESBL production in *Klebsiella pneumoniae* ranged from 55.6 to 88.9%. The resistance of *E. coli* strains to carbapenems was 5.0%, but the overall prevalence of carbapenem-resistant *K. pneumoniae* (CRKP) was high (54.5%).

**Conclusions:**

*S. epidermidis*, *E. coli* and *S. pneumoniae* were the predominant pathogens causing PBM in Chinese patients. The distribution of PBM causative organisms varied by age. The resistance of CoNS to methicillin and the high incidence of ESBL production among *E. coli* and *K. pneumoniae* isolates were concerning. CRKP poses a critical challenge for the treatment of PBM.

## Background

Pediatric bacterial meningitis (PBM) is a devastating infectious disease that causes substantial neurological morbidity and mortality worldwide [1]. Studies have estimated that the annual incidence of PBM in China fluctuated between 7.0 and 22.3 cases per 100 000 children under 5 years old [2]. The case fatality rate was estimated to be 3.0–10.6% [3–5], and survivors had relatively high percentages of neurological complications [3, 5, 6]. Therefore, early diagnosis and prompt use of rational antimicrobial therapies are vital to alleviate the burdens of this disease.

The options of empirical antimicrobial agents in clinical practice mainly depend on the causative organisms and their antimicrobial resistance (AMR) patterns. Immediate and effective antibiotic use for pediatric populations with PBM is relevant for achieving a favorable outcome [7]. However, the spectrum of pathogens causing PBM varies in different reports [8–11]. Data from England and Wales during 2004–2011 indicated that the predominant organisms responsible for PBM were *Neisseria meningitidis* (*N. meningitidis*)*, Streptococcus pneumoniae* (*S. pneumoniae*)*,* and *Staphylococcus aureus* (*S. aureus*) [8]. Meningitis surveillance during 2011–2016 in African regions demonstrated that the two leading causative agents identified were *S. pneumoniae* and *Haemophilus influenzae* (*H. influenzae*) [9, 10]. One study from the southwestern province of China reported that approximately 46.0% of PBM cases were caused by *Escherichia coli* (*E. coli*) and *S. pneumoniae* from 2012 to 2015 [11]. The etiology of PBM varies greatly based on several factors, including geography, time, and patient age [8, 11–13]. In addition to their isolation rates, their AMR patterns vary substantially [14]. Therefore, timely analysis and reporting of the causative agents of PBM and the AMR profiles play vital roles in helping physicians choose the proper empirical therapy.

To the best of our knowledge, there are limited large-scale studies examining the pathogens of PBM in China. Data on the susceptibility patterns of prevailing causative bacteria in pediatric populations are also lacking in China. To gain a better understanding of evidence-based empirical antibiotic treatment and infection control for children with PBM, the present multicenter retrospective survey investigated the features of the etiological agents responsible for PBM and characterized their AMR patterns in China from 2016 to 2018.

## Methods

### Study design and participating hospitals

**Fig. 1:**
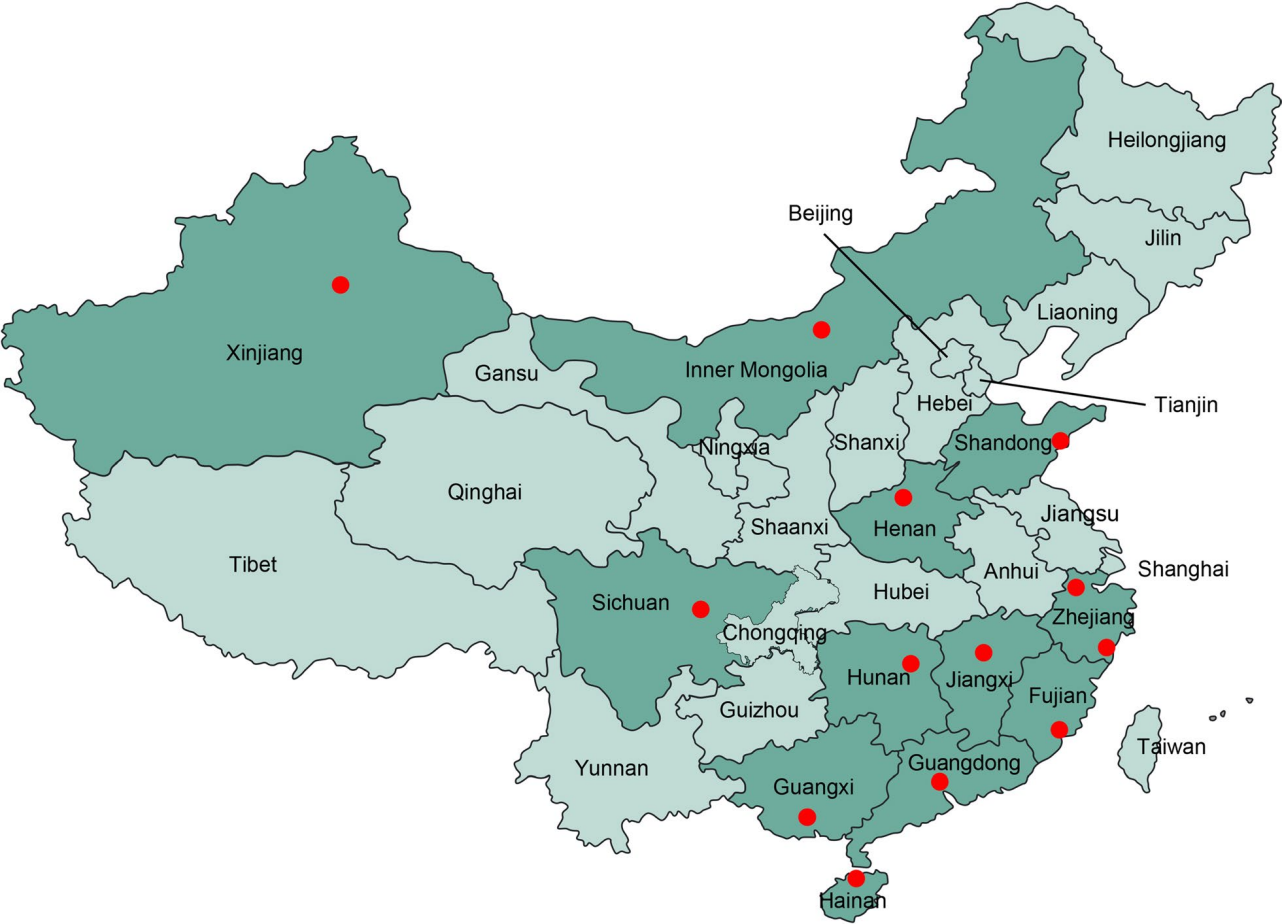
Locations of participating hospitals (red dots) in this study

This national multicenter survey was based on the diagnosis of bacterial meningitis (BM) by cerebrospinal fluid (CSF) culture and was performed retrospectively in 13 children’s hospitals (11 tertiary hospitals and 2 secondary hospitals) in China from January 2016 to December 2018. Thirteen hospitals in 12 provinces provided data for our research. Most of these hospitals were the largest local medical institutions that provided treatment, health consultation or other clinical services for children and/or women. The geographic locations of the participating hospitals are shown in Fig. 1. All participating hospitals have adequate facilities and qualified specialists to conduct bacterial culture and assess antimicrobial susceptibility.

### Study population

Children (< 18 years of age) with BM were enrolled at each hospital based on the review of CSF microbiology results. Patients with clinical manifestations and a positive CSF culture were diagnosed with confirmed BM on the basis of the World Health Organization (WHO) case definition [15]. All eligible patients received antibiotic therapies against target CSF bacterial isolates according to the attending physician’s comprehensive assessment [16]. The exclusion criteria were tuberculous meningitis and fungal meningitis. Duplicate isolates from the same child during hospitalization were not included in the present research. Locally trained researchers collected the data of the enrolled patients from the participating hospital computer data systems, which included the date of sample collection, type of specimen, hospital ward, basic information on patient demographics, causative organisms, antimicrobial susceptibility testing results, and final diagnosis.

### Microbiological methods

All participating hospitals strictly complied with the standard operating procedures for CSF collection and culture. According to the Clinical Laboratory Standards Institute (CLSI) guidelines, local experienced laboratory members of each hospital independently completed the isolation and identification of isolates, and antibiotic susceptibility testing was performed using semiautomated or automated systems. The CLSI guideline was used as the standard for the interpretation of the antimicrobial susceptibility test results [17]. Isolates showing intermediate resistance or resistance to the tested antimicrobial agents were categorized as resistant. The resistance of isolates to carbapenems referred to nonsusceptibility to one or more of the three carbapenems [ertapenem (ETP), imipenem (IMP) and meropenem (MEM)].

### Statistical analysis

We calculated the counts and percentages for categorical variables and analyzed them using the chi-squared test. Continuous variables are presented as medians with the interquartile range. All of the data were collected, stored and sorted in a Microsoft Excel workbook. We performed data analyses using SPSS 22.0 (IBM Corp., New York, NY, USA) software.

## Results

### Demographic characteristics

From January 1, 2016 to December 3, 2018, 1161 patients (< 18 years old) with PBM were enrolled for screening. In accordance with the exclusion criteria, 1142 confirmed PBM patients were ultimately included. Less than half of the enrolled patients were boys (501/1142), and the median age was 3 months (range: 0 days to 17 years, 7 months). Up to 65.9% of patients were < 1 year old, 44.9% were < 3 months old, and a sharp decline in the percentage was observed with increasing age.

### Pathogen composition

A total of 1193 bacterial pathogens were obtained from 1142 patients. Gram-positive organisms accounted for nearly 69.6% (830/1193) of the isolates, and 30.4% (363/1193) were gram-negative organisms. The three leading pathogens causing PBM were *Staphylococcus epidermidis* (*S. epidermidis*) (197/1193; 16.5%), *E. coli* (148/1193; 12.4%) and *S. pneumoniae* (127/1193; 10.6%), followed by *Enterococcus faecium* (*E. faecium*) (115/1193; 9.6%), *Staphylococcus hominis* (*S. hominis*) (85/1193; 7.1%), group B Streptococcus (GBS) (59/1193; 4.9%), *Staphylococcus haemolyticus* (*S. haemolyticus*) (42/1193; 3.5%), *Klebsiella pneumoniae* (*K. pneumoniae*) (40/1193; 3.4%), *S. aureus* (39/1193; 3.3%), and *Acinetobacter baumannii* (*A. baumannii*) (27/1193; 2.3%) (Table 1).

**Table 1:** Common bacterial pathogens isolated from PBM patients in China, 2016–2018

Pathogen	2016 N (%)	2017 N (%)	2018 N (%)	Total N (%)	Rank
Gram-positive organisms					
*Staphylococcus epidermidis*	72 (17.4)	75 (16.7)	50 (15.1)	197 (16.5)	1
*Streptococcus pneumoniae*	41 (9.9)	44 (9.8)	42 (12.7)	127 (10.6)	3
*Enterococcus faecium*	46 (11.1)	32 (7.1)	37 (11.1)	115 (9.6)	4
*Staphylococcus hominis*	15 (3.6)	39 (8.7)	31 (9.3)	85 (7.1)	5
Group B Streptococcus	20 (4.8)	22 (4.9)	17 (5.1)	59 (4.9)	6
*Staphylococcus haemolyticus*	20 (4.8)	11 (2.5)	11 (3.3)	42 (3.5)	7
*Staphylococcus aureus*	16 (3.9)	13 (2.9)	10 (3.0)	39 (3.3)	9
Other gram-positive organisms	56 (13.6)	72 (16.1)	40 (12.1)	168 (14.1)	
Gram-negative organisms					
*Escherichia coli*	49 (11.9)	64 (14.3)	35 (10.5)	148 (12.4)	2
*Klebsiella pneumoniae*	13 (3.1)	10 (2.2)	17 (5.1)	40 (3.4)	8
*Acinetobacter baumannii*	10 (2.4)	12 (2.7)	5 (1.5)	27 (2.3)	10
*Pseudomonas aeruginosa*	6 (1.5)	12 (2.7)	4 (1.2)	22 (1.8)	11
Other gram-negative organisms	49 (11.9)	42 (9.4)	33 (9.9)	124 (10.4)	
Total	413 (34.6)	448 (37.6)	332 (27.8)	1193 (100)	

PBM, pediatric bacterial meningitis

### Distribution of major PBM pathogens according to age and clinical ward

**Fig. 2:**
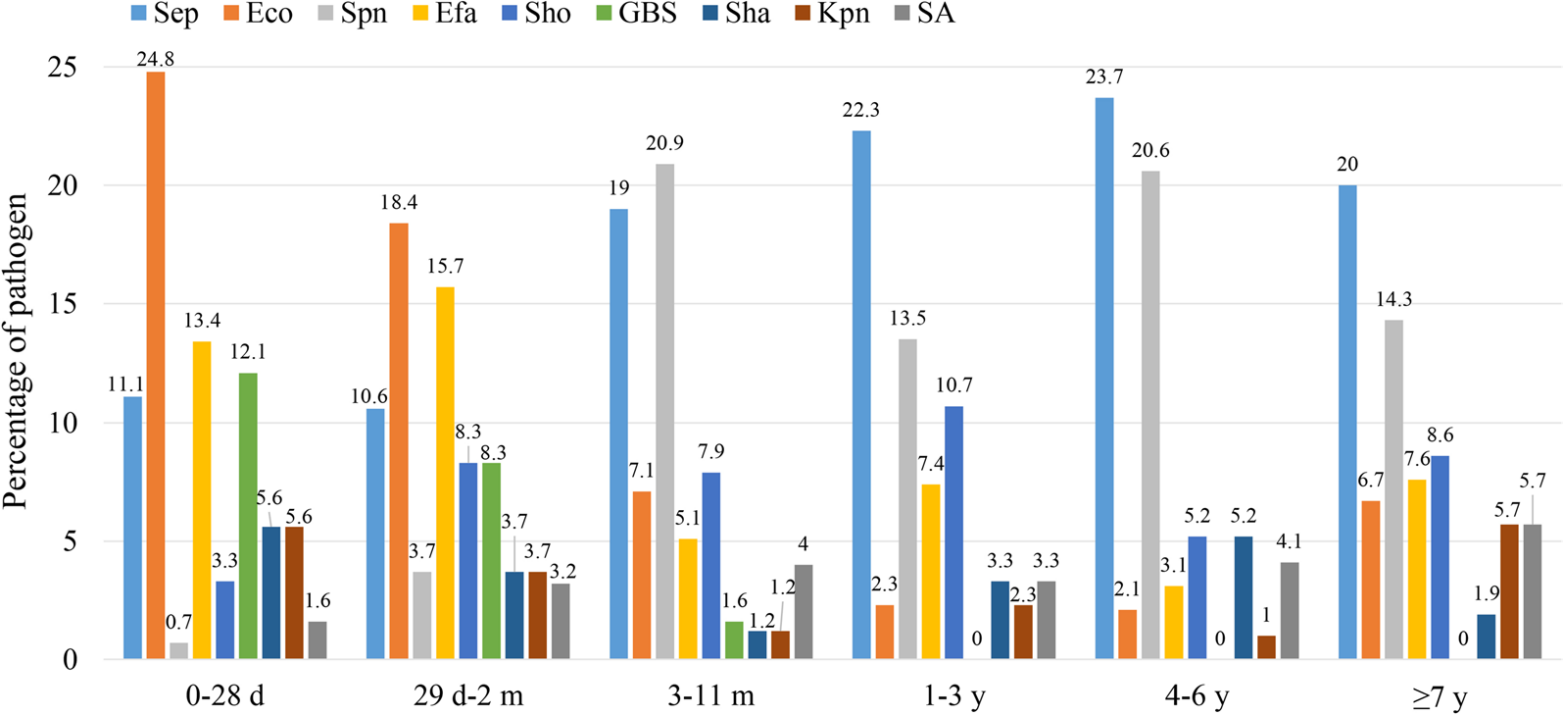
Distribution of major PBM pathogens by age in China, 2016–2018. PBM, pediatric bacterial meningitis; Sep, *Staphylococcus epidermidis*; Eco, *Escherichia coli*; Spn, *Streptococcus pneumoniae*; Efa, *Enterococcus faecium*; Sho, *Staphylococcus hominis*; GBS, group B Streptococcus; Sha, *Staphylococcus haemolyticus*; Kpn, *Klebsiella pneumoniae*; SA, *Staphylococcus aureus*

The spectrum of pathogens causing PBM was highly variable in children of different ages (Fig. 2). In infants under 3 months of age, the top 3 pathogens were *E. coli* (116/523; 22.2%), *E. faecium* (75/523; 14.3%), and *S. epidermidis* (57/523; 10.9%). However, in children more than 3 months of age, the top 3 pathogens were *S. epidermidis* (140/670; 20.9%), *S. pneumoniae* (117/670; 17.5%), and *S. hominis* (57/670; 8.5%). As shown in Fig. 2, the other prevalent bacteria also varied by age group. Children less than 1 year old had the greatest abundance of pathogenic species, the leading pathogen among which was *E. coli* (134/776; 17.3%), followed by *S. epidermidis* (105/776; 13.5%) and *E. faecium* (88/776; 11.3%).

In terms of clinical ward distribution, these common pathogens were detected in the pediatric intensive care unit (PICU), surgical department and other departments (Additional file 1: Figure S1). *S. epidermidis* was detected more commonly in the surgical department (31.1%) than in the PICU (10.8%) and other departments (13.9%) (*P* < 0.001). However, *E. coli, S. pneumoniae, E. faecium,* and GBS were detected more frequently in the PICU and in the other departments than in the surgical department (all *P* < 0.05).

### AMR patterns of the major gram-positive bacteria

**Fig. 3:**
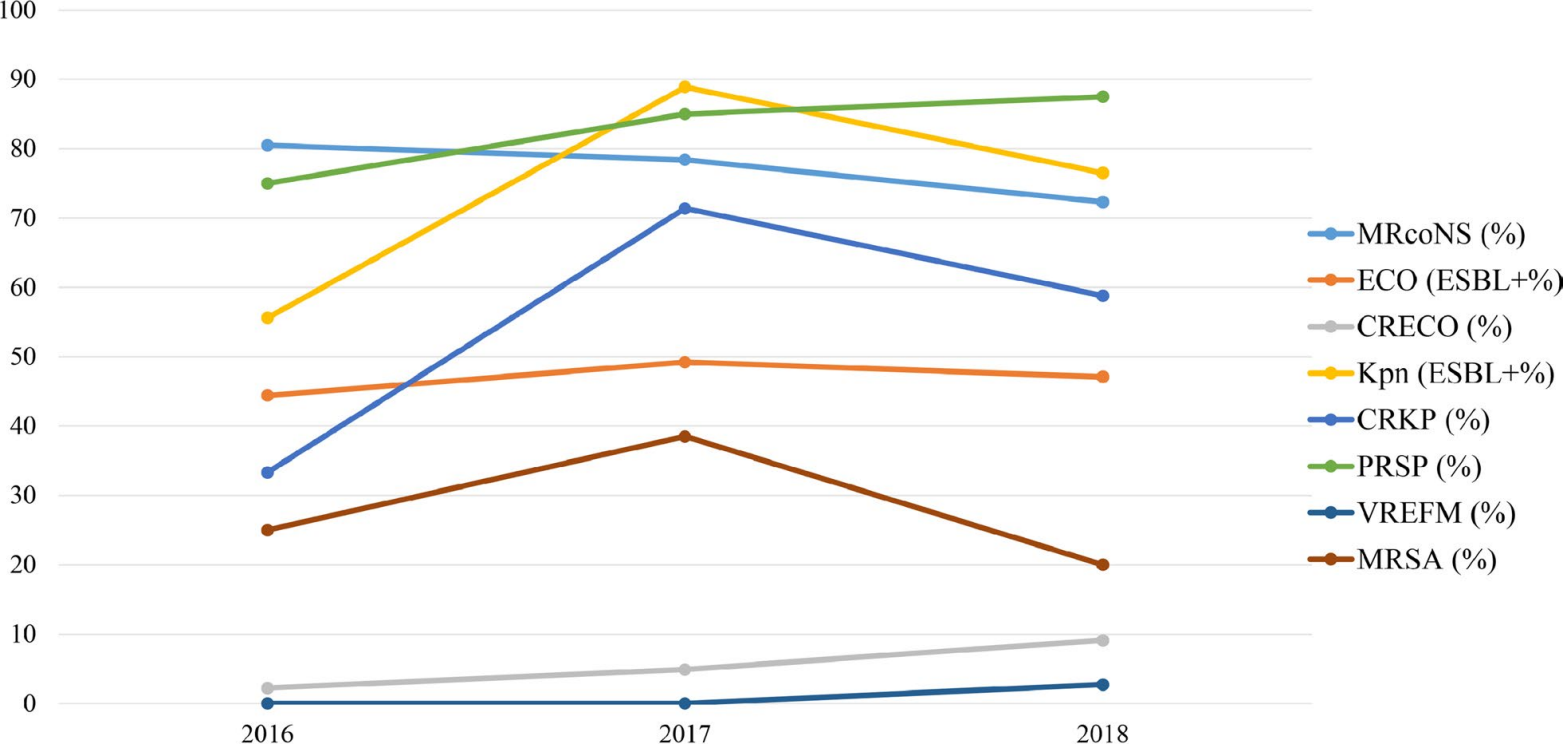
Trends of common multidrug resistant strains isolated from PBM patients in China, 2016–2018. PBM, pediatric bacterial meningitis; MRCoNS, methicillin-resistant coagulase-negative Staphylococcus; ESBL, extended-spectrum β-lactamase; CRECO, carbapenem-resistant *Escherichia coli*; CRKP, carbapenem-resistant *Klebsiella pneumoniae*; PRSP, penicillin-resistant *Streptococcus pneumonia*e; VREFM, vancomycin-resistant *Enterococcus faecium*; MRSA, methicillin-resistant *Staphylococcus aureus*

As shown in Table 2, the three main species of coagulase-negative Staphylococcus (CoNS)*,* namely, *S. epidermidis, S. hominis* and *S. haemolyticus*, were isolated from CSF cultures. The resistances of these isolates to penicillin (PEN) and erythromycin (ERY) were greater than 71.0%. Resistance to methicillin depended on oxacillin (OXA) resistance. Therefore, the overall detection rate of the methicillin-resistant CoNS (MRCoNS) isolates was approximately 80.0% and declined from 80.5% in 2016 to 72.3% in 2018 (Fig. 3). All of these three species were susceptible to linezolid (LNZ) (100%) and vancomycin (VAN) (100%). Over 65.0% of the isolates, except *S. haemolyticus*, were also susceptible to aminoglycosides, fluoroquinolones, co-trimoxazole (SXT), rifampin (RIF) and tetracycline (TET). For *S. pneumoniae* isolates, resistance to fluoroquinolones, LNZ, or VAN was not detected in this study. The susceptibility to amoxicillin (AMX), cefotaxime (CTX) and ceftriaxone (CRO) was 74.8%, 59.0%, and 50.0%, respectively. However, over 84.0% of the *S. pneumoniae* isolates were resistant to ERY, clindamycin (CLI), SXT, and TET. The resistance of *S. pneumoniae* isolates to PEN was 82.8%, increasing from 75.0% in 2016 to 87.5% in 2018 (Fig. 3). More than 95.0% of *E. faecium* isolates showed susceptibility to VAN, LNZ, and tigecycline (TGC), but the resistance rates to other antibacterial drugs were over 62.9%.

**Table 2:** Resistance rates (%) of major gram-positive bacteria of PBM patients in China, 2016–2018

Antimicrobial agent	Sep A/B (%)	Sho A/B (%)	Sha A/B (%)	Spn A/B (%)	Efa A/B (%)	GBS A/B (%)	SA A/B (%)
Oxacillin	132/165 (80.0)	51/67 (76.1)	31/36 (86.1)	–	–	–	9/31 (29.0)
Penicillin G	143/159 (89.9)	58/66 (87.9)	28/33 (84.8)	92/116 (82.8)	90/92 (97.8)	1/58 (1.7)	1/29 (96.6)
Amoxicillin	–	–	–	26/103 (25.2)	–	–	–
Ampicillin	–	–	–	–	106/111 (95.5)	1/27 (3.7)	–
Cefotaxime	–	–	–	48/117 (41.0)	–	–	–
Ceftriaxone	–	–	–	17/34 (50.0)	–	–	–
Erythromycin	134/160 (83.8)	50/70 (71.4)	35/36 (97.2)	112/116 (96.6)	107/109 (98.2)	28/31 (90.3)	22/31 (71.0)
Clindamycin	–	–	–	60/65 (92.3)	–	45/49 (91.8)	9/25 (36.0)
Gentamicin	44/163 (27.0)	14/69 (20.3)	23/36 (63.9)	–	–	–	2/31 (6.5)
Ciprofloxacin	46/165 (27.9)	15/69 (21.7)	30/36 (83.3)	–	102/110 (92.7)	–	1/32 (3.2)
Moxifloxacin	29/94 (30.9)	13/55 (23.6)	10/17 (58.8)	0/78 (0)	–	–	0/19 (0)
Levofloxacin	30/98 (30.6)	8/58 (13.8)	10/19 (52.6)	0/118 (0)	58/65 (89.2)	28/59 (47.5)	2/21 (9.5)
Rifampin	27/163 (16.6)	8/66 (12.1)	12/35 (34.3)	–	–	–	1/31 (3.2)
Nitrofurantoin	–	–	–	–	66/105 (62.9)	–	0/30 (0)
Co-trimoxazole	57/164 (34.8)	17/67 (25.4)	30/36 (83.3)	101/119 (84.9)	–	–	8/31 (25.8)
Tetracycline	45/165 (27.3)	19/68 (27.9)	14/37 (37.8)	107/117 (91.5)	83/111 (74.8)	33/48 (68.8)	7/32 (21.9)
Vancomycin	0/164 (0)	0/71 (0)	0/37 (0)	0/120 (0)	1/111 (0.9)	0/50 (0)	0/31 (0)
Linezolid	0/192 (0)	0/82 (0)	0/41 (0)	0/94 (0)	5/114 (4.4)	0/56 (0)	0/38 (0)
Tigecycline	–	–	–	–	0/63 (0)	–	–

PBM, pediatric bacterial meningitis; Sep, *Staphylococcus epidermidis*; Sho, *Staphylococcus hominis*; Sha, *Staphylococcus haemolyticus*; Spn, *Streptococcus pneumoniae*; Efa, *Enterococcus faecium*; GBS, group B Streptococcus; SA, *Staphylococcus aureus*

A/B (%), number resistant/number tested (percentage resistant)

A dash (–) indicates that antibiotics were not tested against the isolated pathogens

### AMR patterns of the major gram-negative bacteria

As shown in Table 3, more than 93.0% of the *E. coli* isolates were sensitive to cefoxitin (FOX), piperacillin/tazobactam (TZP), cefoperazone/sulbactam (CSL), amikacin (AMK) and carbapenems, and the resistance rates for ampicillin (AMP) and piperacillin (PIP) exceeded 82.0%. The sensitivities of *E. coli* to third-generation cephalosporins, aztreonam and fluoroquinolones were 47.6–73.6%, 65.3%, 44.3–46.4%, respectively. Moreover, the detection rate of ESBL-producing *E. coli* was stable and fluctuated between 44.4 and 49.2% in 2016–2018. The proportion of carbapenem-resistant *E. coli* (CRECO) isolates was 5.0%, increasing from 2.2% in 2016 to 9.1% in 2018 (Fig. 3). *K. pneumoniae* isolates exhibited susceptibility rates greater than 50.0% to aminoglycosides, fluoroquinolones, TET, SXT, IMP, and ETP, but the resistance rates to other antibiotics were greater than 50.0%. The proportions of ESBL-producing *K. pneumoniae* and carbapenem-resistant *K. pneumoniae* (CRKP) were 74.3% and 54.5%, respectively. The data from 2016 to 2018 demonstrated that ESBL-producing *K. pneumoniae* and CRKP showed an upward trend (Fig. 3).

**Table 3:** Resistance rates (%) of major gram-negative bacteria of PBM patients in China, 2016–2018

Antimicrobial agent	Eco A/B (%)	Kpn A/B (%)	Aba A/B (%)	Pae A/B (%)
Ampicillin	105/125 (84.0)	25/26 (96.2)	14/18 (77.8)	12/13 (92.3)
Piperacillin	56/68 (82.4)	11/14 (78.6)	3/5 (60.0)	5/13 (38.5)
Cefazolin	52/104 (50.0)	18/23 (78.3)	–	–
Cefuroxime	11/21 (52.4)	4/8 (50.0)	–	–
Cefoxitin	0/30 (0)	5/8 (62.5)	–	–
Ceftriaxone	44/89 (49.4)	10/15 (66.7)	8/15 (53.3)	–
Cefotaxime	30/61 (49.2)	14/20 (70.0)	5/8 (62.5)	–
Ceftazidime	38/140 (26.4)	20/32 (62.5)	8/21 (38.1)	5/16 (31.2)
Cefepime	45/140 (32.1)	17/32 (53.1)	7/21 (33.3)	3/17 (17.6)
Amoxicillin/clavulanic acid	–	–	8/13 (61.5)	–
Ampicillin/Sulbactam	58/102 (56.9)	20/26 (76.9)	5/15 (33.3)	7/7 (100)
Piperacillin/tazobactam	3/122 (2.5)	20/30 (66.7)	5/12 (41.7)	4/10 (40.0)
Cefoperazone/sulbactam	1/17 (5.9)	–	–	–
Tobramycin	23/56 (41.1)	2/8 (25.0)	1/10 (10.0)	¼ (25.0)
Gentamicin	49/127 (38.6)	13/30 (43.3)	5/19 (41.7)	2/13 (15.4)
Amikacin	0/142 (0)	11/31 (35.5)	2/16 (12.5)	1/16 (6.2)
Ciprofloxacin	73/131 (55.7)	12/29 (41.4)	5/20 (25.0)	1/13 (7.7)
Levofloxacin	74/138 (53.6)	12/32 (37.5)	5/21 (23.8)	3/16 (18.8)
Aztreonam	43/124 (34.7)	17/26 (65.4)	–	2/4 (50.0)
Tetracycline	60/76 (78.9)	9/30 (30.0)	5/9 (55.6)	–
Co-trimoxazole	97/141 (68.8)	5/34 (14.7)	13/22 (59.1)	–
Meropenem	6/88 (6.8)	16/25 (64.0)	6/11 (54.5)	2/13 (15.4)
Ertapenem	0/79 (0)	1/12 (8.3)	–	–
Imipenem	4/140 (2.9)	5/22 (22.7)	6/19 (31.6)	6/16 (37.5)

PBM, pediatric bacterial meningitis; Eco, *Escherichia coli*; Kpn, *Klebsiella pneumoniae*; Aba, *Acinetobacter baumannii*; Pae, *Pseudomonas aeruginosa*

A/B (%), number resistant/number tested (percentage resistant)

A dash (–) indicates that antibiotics were not tested against the isolated pathogens

For *A. baumannii* and *P. aeruginosa* isolates, the susceptibility rates of *A. baumannii* isolates to aminoglycosides and fluoroquinolones exceeded 55.0%. The susceptibility of isolates to cephalosporins and carbapenems ranged from 37.5 to 66.7% and 45.5 to 68.4%, respectively. The susceptibility rates of *P. aeruginosa* isolates to PIP, third-generation cephalosporins, TZP, aminoglycosides, fluoroquinolones, and carbapenems were greater than 60.0%, but the resistance of isolates to AMP and ampicillin/sulbactam (SAM) exceeded 92.0%.

## Discussion

PBM remains a serious threat to children’s health worldwide despite significant progress in the diagnosis and treatment of the disease. In the present study, *S. epidermidis* (16.5%) was the leading causative pathogen, followed by *E. coli* (12.4%) and *S. pneumoniae* (10.6%). Our findings differed from various reports worldwide, including reports from Britain [8], Africa [9, 10] and the USA [18], which showed that *N. meningitidis, S. pneumoniae* and *H. influenzae* were the predominant causative agents of PBM. However, the results were similar to those of a report from Southwest China [11]. The reasons for the discrepancies between studies are most likely differences in socioeconomic status and environmental factors among these regions. Therefore, timely summary and reporting of the features of the etiological agents of local PBM are urgently needed.

The distribution of PBM pathogens is highly variable in children of different ages [11–13]. We noticed that in infants aged under 3 months, the top 3 pathogens were *E. coli* (22.2%), *E. faecium* (14.3%), and *S. epidermidis* (10.9%). This result was similar to previous Chinese reports from Yunnan [11] and Shanghai [19]. However, it was not in accordance with several studies from the USA [20] and the UK and Ireland [21], which showed that GBS, *E. coli* and *S. aureus* were the predominant pathogens causing PBM in infants younger than 3 months. We also observed that in children older than 3 months of age, the predominant organisms causing PBM were *S. epidermidis* (20.9%), *S. pneumoniae* (17.5%) and *S. hominis* (8.5%). This finding was consistent with a previous study conducted in mainland China [11] but differed from data from England and Wales [8]. In addition, we also found that children younger than 1 year old had the highest abundance of pathogenic species, the leading pathogen among which was *E. coli*, followed by *S. epidermidis* and *E. faecium*. Therefore, the options of empiric antimicrobial agents for patients with PBM should be stratified by age to cover the most likely organisms.

The resistance rates of CoNS isolates to PEN and ERY were greater than 71.0% in the present study, which was similar to previous studies conducted in Brazil (100% and 86.2%, respectively) [22] and Beijing, China (94.9% and 92.4%, respectively) [23]. The overall resistance of the CoNS species to methicillin was nearly 80.0%, and the proportion of MRCoNS isolates declined slightly over the 3 years but remained very high (Fig. 3). These surveillance data were in line with a report from Beijing, China (93.6%) [23] but higher than the values reported in eastern Nepal (57.7%) [24] and Ethiopia (37.0%) [25]. Consistent with most previous studies [22, 24, 25], there were no LNZ- or VAN-resistant isolates found in our study. VAN remained the best option for the treatment of MRCoNS infections in patients with PBM. However, data from the UK and Ireland during 2013–2015 demonstrated that 0.2% of CoNS isolates were resistant to methicillin [26]. VAN-resistant CoNS exists in the clinic. Although no VAN-resistant CoNS isolates were found in these surveillance data, the emerging resistance of CoNS isolates to VAN may be a serious concern in PBM.

The high resistance of *E. coli* and *K. pneumoniae* to antibiotics is of critical concern [27–31]. The resistance rates of *E. coli* to CRO (49.4%) and CTX (49.2%) in the present study were quite close to those in previous Chinese studies [11, 13]. Our research also found that the proportion of ESBL-producing *E. coli* fluctuated between 44.4 and 49.2% in 2016–2018, which was in line with the proportions reported in Pakistan (40.0%) [27] and East Africa (42.0%) [28] but higher than the proportion in the USA (ranging from 10.0 to 15.0%) [29]. Notably, the proportion of CRECO was 5.0%, and this increased from 2.2% in 2016 to 9.1% in 2018. Data from the CHINET surveillance in 2005–2014 reported that IMP and MEM resistance rates of *E. coli* were approximately 1.0% and 2.2%, respectively [30]. A survey in the USA showed that the detected rates of CRECO increased from 2001 to 2010 [31]. However, the incidence of ESBL production in *K. pneumoniae* isolates and the resistance rates of *K. pneumoniae* to cephalosporins, aminoglycosides and carbapenems were higher than those of the *E. coli* isolates in our research. The prevalence of CRKP was 54.5% during the 3 years of this study. Consistent with this result, there were distinct upward trends of CRKP rates in 5 of the 24 participating countries in Europe from 2009 to 2012. Approximately 60.0% of *K. pneumoniae* isolates were resistant to carbapenems in Greece in 2012 [32]. Previous studies showed that carbapenem-resistant Enterobacteriaceae strains caused a mortality rate of approximately 26.0–44.0% [33], prolonged hospitalization time and increased economic burdens on patients [34]. Carbapenems were proposed as the optimal treatment for carbapenem-resistant Enterobacteriaceae infections. However, resistance to carbapenems has become an extreme challenge in the treatment of CRKP. Additional studies should focus on the mechanism of CRKP production in the future.

We also observed that the resistance rate of *S. pneumoniae* isolates to PEN was 82.8%, and the ratio increased from 75.0% in 2016 to 87.5% in 2018, which was lower than the values reported in Beijing (95.7%) [35] and Ethiopia (100%) [36] but was higher than the values from other studies, including those in Yunnan (31.2%) [11] and Iran (21.0%) [37]. *S. pneumoniae* had resistance rates of 41.0% to CTX and 50.0% to CRO in the present study. A finding from Beijing showed that nearly 14.0% and 11.3% of *S. pneumoniae* isolates were resistant to CTX and CRO, respectively, from 2014 to 2016 [13]. Jiang et al. [11] found that 16.7% of *S. pneumoniae* isolates were resistant to CRO in Yunnan during 2012–2015. The Ethiopian study indicated that the CTX and CRO resistance rates of *S. pneumoniae* isolates were 60.0% and 60.0%, respectively [36]. Various resistance levels were observed in different reports, and the high incidence of resistance in our study may be ascribed to the greater exposure to antibiotics in the clinic. Therefore, the use of antibiotics deserves serious consideration for the reduction in antibiotic resistance in China. Not surprisingly, there were no LNZ- or VAN-resistant *S. pneumoniae* isolates found in our study, which coincided with previous studies [11–13, 36, 37].

The present study was the first large-scale study to summarize the features of the etiological agents and AMR profiles of PBM in China. However, several limitations exist. First, due to the lack of patients’ detailed clinical data, we were unable to distinguish community- and hospital-acquired PBM or assess the epidemiological differences between them. We were also unable to identify a relationship between hospital-acquired PBM and pathogen distribution by clinical wards. Second, although all of the antimicrobial resistance results were interpreted based on the CLSI guideline, variations were inevitably found in the detection platforms, automated systems and technical skills of the participating hospitals, and these variations may have affected the results of the antimicrobial sensitivity tests to some degree.

## Conclusions

The present study identified that *S. epidermidis*, *E. coli* and *S. pneumoniae* were the three leading bacterial pathogens associated with PBM in China from 2016 to 2018. It also revealed that the distribution of common PBM causative organisms varied by age. The resistance of CoNS to methicillin and the high incidence of ESBL production in *E. coli* and *K. pneumoniae* were concerning. CRKP poses a critical challenge for PBM treatment. Therefore, enhancing the surveillance and timely reporting of antibiotic resistance is urgently needed in China.

## Supplementary Information

**Additional file 1: Figure S1.** Distribution of major PBM pathogens according to clinical wards in China, 2016–2018. PBM: pediatric bacterial meningitis; Sep, *Staphylococcus epidermidis*; Eco, *Escherichia coli*; Spn, *Streptococcus pneumoniae*; Efa, *Enterococcus faecium*; Sho, *Staphylococcus hominis*; GBS, group B Streptococcus; Sha, *Staphylococcus haemolyticus*; Kpn, *Klebsiella pneumoniae*; SA, *Staphylococcus aureus.*

## Data Availability

The datasets in the present study are accessible from the corresponding author on reasonable request.

## Abbreviations

AMR: Antimicrobial resistance
AMP: Ampicillin
AMC: Amoxicillin/clavulanic acid
ATM: Aztreonam
AMK: Amikacin
CZO: Cefazolin
CXM: Cefuroxime
CLSI: Clinical and Laboratory Standards Institute
CAZ: Ceftazidime
CTX: Cefotaxime
CLI: Clindamycin
CHL: Chloramphenicol
CIP: Ciprofloxacin
CoNS: Coagulase-negative Staphylococcus
CRO: Ceftriaxone
CSL: Cefoperazone/sulbactam
CRE: Carbapenem-resistant Enterobacteriaceae
CRECO: Carbapenem-resistant *Escherichia coli*
CRKP: Carbapenem-resistant *Klebsiella pneumonia*
ETP: Ertapenem
ERY: Erythromycin
ESBL: Extended-spectrum β-lactamase
Efa: *Enterococcus faecium*
Eco: *Escherichia coli*
FEP: Cefepime
GBS: Group B Streptococcus
GEN: Gentamicin
IMP: Imipenem
Kpn: *Klebsiella pneumonia*
LNZ: Linezolid
LVX: Levofloxacin
MRCoNS: Methicillin-resistant coagulase-negative Staphylococcus
MXF: Moxifloxacin
MEM: Meropenem
MRSA: Methicillin-resistant *Staphylococcus aureus*
OXA: Oxacillin
RIF: Rifampin
PEN: Penicillin G
PIP: Piperacillin
PBM: Pediatric bacterial meningitis
PRSP: Penicillin-resistant *Streptococcus pneumonia*
Sep: *Staphylococcus epidermidis*
Spn: *Streptococcus pneumonia*
Sho: *Staphylococcus hominis*
Sha: *Staphylococcus haemolyticus*
SA: *Staphylococcus aureus*
TZP: Piperacillin/tazobactam
TET: Tetracycline
VAN: Vancomycin
VREFM: Vancomycin-resistant *Enterococcus faecium*
