# Supplementary figures and images for: Prevalence and antimicrobial resistance patterns of bacteria isolated from cerebrospinal fluid among children with bacterial meningitis in China from 2016 to 2018: a multicenter retrospective study
Source: Antimicrob Resist Infect Control. 2021 Jan 30;10:24. doi: 10.1186/s13756-021-00895-x (PMC7847565; doi:10.1186/s13756-021-00895-x)

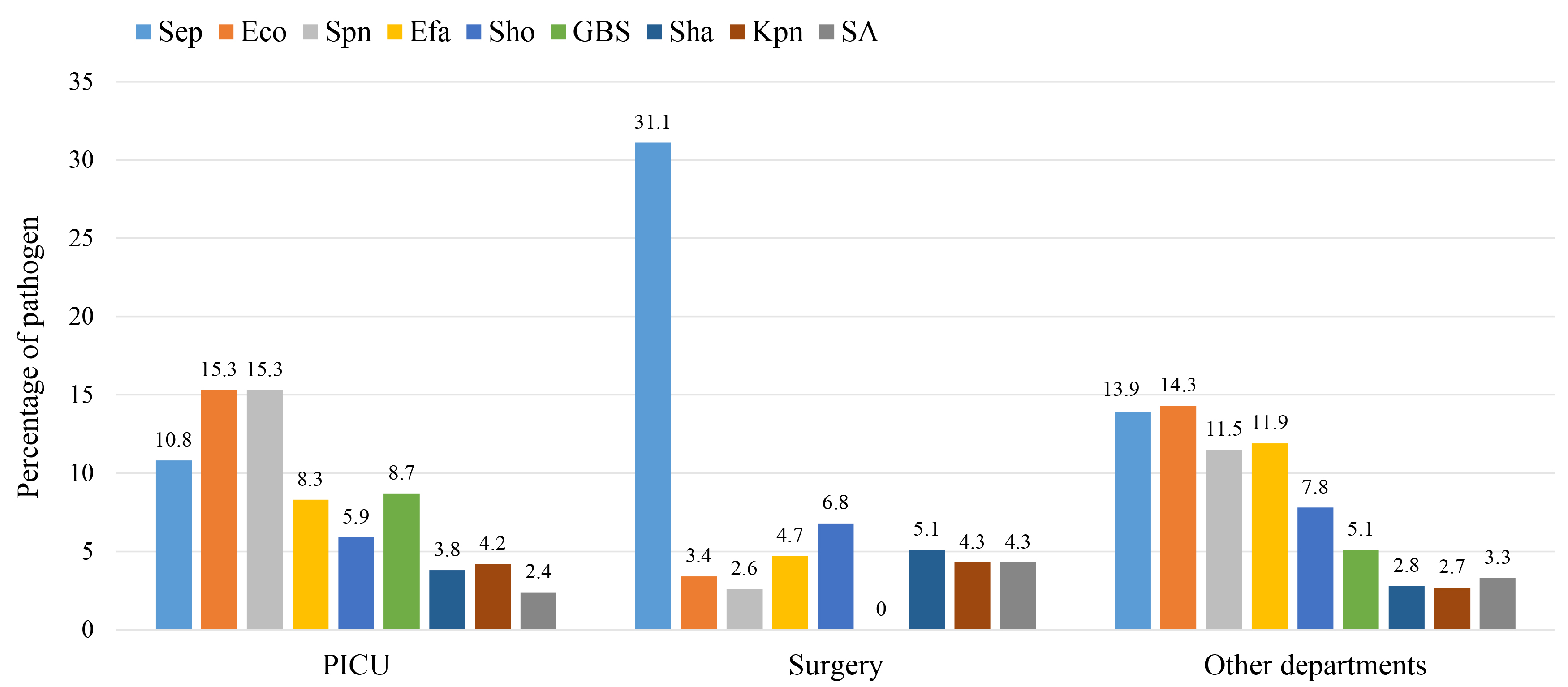

Supplement: Supplementary file 1 — Additional file 1: Figure S1. Distribution of major PBM pathogens according to clinical wards in China, 2016–2018. PBM: pediatric bacterial meningitis; Sep, Staphylococcus epidermidis; Eco, Escherichia coli; Spn, Streptococcus pneumoniae; Efa, Enterococcus faecium; Sho, Staphylococcus hominis; GBS, group B Streptococcus; Sha, Staphylococcus haemolyticus; Kpn, Klebsiella pneumoniae; SA, Staphylococcus aureus. [file 13756_2021_895_MOESM1_ESM.jpg]
